# The Treatment of Stenosis of the Larynx and Trachea

**Published:** 1909-04-17

**Authors:** 


### Laryngology and Rhinology.

### THE TREATMENT OF STENOSIS OF THE LARYNX AND TRACHEA.

Obstruction of the larynx and trachea is produced by so great a multiplicity of causes, from inflammatory swelling to the impaction of foreign bodies, that a complete discussion of the treatment is beyond the scope of this column. Indeed, it would be inconvenient to describe the treatment of neoplasms and of foreign bodies under this heading, and our remarks will only be directed to certain points.

In all forms of stenosis, when dyspnœa is severe and urgent the imperative indication is to relieve it by tracheotomy, laryngotomy, or intubation. Laryngotomy has the advantage that it can be very rapidly and easily performed, and is therefore a suitable procedure when suffocation is extreme and allows no time for any preparation; a knife only is required, and the wound can be kept open with a hairpin or any similar object. It is the operation of choice when the dyspnœa is due to the impaction of a foreign body in the larynx or to fracture of the thyroid cartilage, for the incision may, if necessary, be extended into a laryngo-fissure; it is also a very convenient method of opening the air-passage before any extensive operation about the mouth or pharynx. But it is unsuitable for young children, in whom the crico-thyroid space is too small, and it should never be performed when the tube is to be retained for more than a very few hours, for stenosis is then apt to follow. There has been much discussion as to the relative advantages of tracheotomy and intubation, which latter operation is practised more extensively abroad than in this country. Intubation is in general suited only to cases of temporary obstruction, and practically to cases of diphtheria; it is quite unsuitable for such forms of disease as are accompanied by deep ulceration and inflammation, such as perichondritis, tuberculosis, or active syphilis. It can, however, be successfully employed in the treatment of cicatricial stenosis. On account of the risk of the tube being coughed out, it should be used in diphtheria only when skilled attention is at hand; but, with this proviso, it is probably a safer method than tracheotomy in young children.

When dyspnœa is caused by œdema supervening in a case of acute laryngitis, perichondritis, etc., the necessity for tracheotomy may often be obviated by timely free scarification of the swollen aryteno-epiglottic folds. In children this is best done with a curved bistoury, protected nearly to the point with strapping, and guided by the left forefinger; in adults the operation is performed with a laryngeal lancet under inspection with the mirror. If tracheotomy is necessary it should be performed low down, as subglottic swelling may be present, and extend for some distance down the trachea. Rarely the swelling of the upper aperture in tuberculous laryngitis may produce dyspnœa; in such cases the patient nearly always sinks rapidly after tracheotomy, but this operation can be avoided by incision, or, better, by partial removal of the swollen tissues with punch-forceps. So also in laryngeal syphilis tracheotomy may be prevented if the patient be rapidly got under the influence of mercury by intramuscular injection; but here it is less important to avoid the operation, for tracheotomy appears to have a distinctly favourable effect on the laryngeal lesions. Difficulty in dispensing with the tube after tracheotomy in children, when due to definite stenosis, is usually the result of damage to the larynx at the time of the operation by making the incision too high, and must be treated as cicatricial stenosis of the larynx; but it is very often merely due to spasm from terror when the tube is taken out, and is then best treated by encouraging the patient to blow soap-bubbles or sound a whistle, using a fenestrated tube, and later stopping the outer opening; sometimes it suffices to remove the tube as though to change it, and to tie only the shield on to the neck. Of a very common cause of tracheal stenosis, pressure by a goître, it may be said here that tracheotomy should be avoided if possible, as it renders a subsequent operation much more difficult, and makes strict asepsis impossible; a cyst may be tapped or an adenoma shelled out, and in a case of parenchymatous goître the dyspnœa may often be relieved by dividing the isthmus; but it is best to remove the goître before the dyspnœa has become dangerously severe. In cases of bilateral abductor paralysis of the cords, an urgent attack of suffocation may come on at any time, and therefore a tracheotomy tube should be permanently worn; it may be closed with a plug, to be removed at night and whenever there is dyspnœa.

It may be said at once that the treatment of cicatricial stenosis is always troublesome and tedious, and demands much ingenuity from the surgeon and much patience from the patient. As in the first instance, tracheotomy is often required, attempts have been made to dilate the stricture from below by means of an attachment fixed to the tube; but these are seldom satisfactory; they are painful, difficult to bear, and apt to produce pressure ulceration. It is generally better to dilate from above. In the first place the stricture must first be thoroughly divided; this is done under cocaine with a Whistler's guarded knife, or, better, at any rate in expert hands, with a probe-pointed laryngeal bistoury, which is introduced below the stricture, and made to cut upwards well into the anterior commissure. Dilatation is kept up by means of Schrötter's bougies, or by intubation tubes. The former are curved hollow vulcanite bougies, triangular in section to fit the glottis; they can be kept in only for a few minutes at a time, and are passed daily, proceeding gradually to larger sizes. After dilatation is complete they are introduced at lengthening intervals, the entire treatment lasting for many months. Intubation tubes are used in the same way, but can be left in for a longer time, even up to twenty-four hours. The thread should be left attached to facilitate removal and to prevent the tube falling into the trachea as the stricture dilates. When the obstruction is very massive it may be advisable to perform thyrotomy, and cut away the scar-tissue, but dilatation will have to be continued afterwards. These cases are most intractable, and should be kept under observation, and, if necessary, dilated at intervals, for several years. In the worst cases "laryngostomy" is recommended, *i.e.* performing thyrotomy, excising the stricture, keeping the wound open and carefully packed for two or three months, and finally closing it by a plastic operation.

Stenosis of the lower part of the trachea, usually syphilitic, is most difficult to treat; as it must otherwise terminate fatally, every effort must be made. The stricture should be dilated through a tracheotomy wound with hollow bougies, and it may now be incised and dilated under direct inspection through the tracheoscope. The only alternative is to use a long flexible cannula after low tracheotomy.

